# Does serial administration of gadolinium‐based contrast agents affect patient neurological and neuropsychological status? Fourteen‐year follow‐up of patients receiving more than fifty contrast administrations

**DOI:** 10.1002/jmri.26948

**Published:** 2019-10-30

**Authors:** Josef Vymazal, Lenka Krámská, Hana Brožová, Evžen Růžička, Aaron M. Rulseh

**Affiliations:** ^1^ Department of Radiology Na Homolce Hospital Prague Czech Republic; ^2^ Department of Neurology Clinical Psychology, Na Homolce Hospital Prague Czech Republic; ^3^ Department of Neurology and Centre of Clinical Neuroscience Charles University, First Faculty of Medicine Prague Czech Republic

To the Editor:

Gadolinium‐based contrast agents (GBCAs) have been in clinical use for ~30 years and have been considered relatively safe in terms of acute allergy‐like and chemotoxic reactions.^1^ The demonstration of increased signal intensity (SI) in the brain, particularly in the deep brain nuclei (dentate nucleus [DN] and globus pallidus [GP]) on unenhanced T_1_‐weighted images following cumulative GBCA dosing and evidence of the presence of gadolinium (Gd) in these nuclei (and elsewhere)^2^ has generated concern over potential long‐term detrimental effects of these agents, potentially leading to severe neurological deficits. As yet, however, no clinical manifestations of Gd toxicity or adverse clinical outcomes related to brain Gd retention have been observed following the repeated administration of any GBCA. Nevertheless, all linear GBCAs have been suspended in Europe with the exception of two substituted linear agents (MultiHance, Bracco Diagnostics, Princeton, NJ; and Primovist, Bayer Healthcare, Berlin, Germany) that are uniquely specific for liver imaging. The rationale for the suspension has been stated as: "to prevent any risks that could potentially be associated with gadolinium brain deposition."^3^

The basal ganglia and DN are primarily involved in cognitive processing and motor control.^4^ Damage to the DN and GP may therefore be expected to result predominantly in movement disorder manifestations such as resting tremor, rigidity, bradykinesia, and gait abnormalities, as well as cognitive impairment, depression, and neurobehavioral deficits. We therefore aimed to assess the neurological and neuropsychological status of four patients who received exceptionally large cumulative doses of GBCAs over many years at our center for the diagnosis and follow‐up of glioblastoma multiforme (GBM).

## Materials and Methods

We performed neurological and neuropsychological evaluation on four patients diagnosed with GBM in 2004–2005, verified histologically at two independent laboratories, who subsequently received at least 50 GBCA administrations as part of routine follow‐up (Table 1). The study was approved by the institutional Ethics Committee and all subjects provided signed, informed consent to participate. Early magnetic resonance imaging (MRI) examinations were performed almost exclusively with Omniscan (GE Healthcare, Milwaukee, WI) and Magnevist (Bayer Healthcare, Berlin, Germany) at monthly intervals. Thereafter, examinations were performed primarily with MultiHance and macrocyclic GBCAs at bimonthly intervals or longer. Patients received fixed GBCA volumes that varied based on GBCA relaxivity and the concentration of the formulation. The mean (±standard deviation) volume of the 0.5 M agents was 10.8 ± 2.3 mL, with overall higher volumes administered for dotarem due to its lower relaxivity. The mean administered volume of the 1 M agent gadovist was 7.3 ± 2.2 mL.

**Table 1:** Summary of Gadolinium‐Based Contrast Agent (GBCA) Administrations

GBCA type	GBCA	Patient 1		Patient 2		Patient 3		Patient 4	
		No. of exams	Total volume (mL)	No. of exams	Total volume (mL)	No. of exams	Total volume (mL)	No. of exams	Total volume (mL)
Simple linear	Omniscan	10	110	15	155	9	90	7	70
	Magnevist	14	140	10	100	9	90	6	60
Substituted linear	MultiHance	22	216	12	134	24	279	23	234
Macrocyclic	Gadovist	10	(56) 112^a^	31	(248) 496^a^	11	(86) 172^a^	9	(52.5) 105^a^
	ProHance	2	19	—	—	1	9	5	59
	Dotarem	6	83	—	—	5	96	1	14
	Unknown^b^	2	20	3	30	—	—	2	20
	Total	66	700	71	915	59	736	53	562

a Volume based on equivalence for a 0.5 M formulation.

b Highly likely to be simple linear GBCAs based on examination dates.

Detailed neurological and neuropsychological evaluations were performed in 2018, ~12–14 years after initial diagnosis. Neurological assessment in 2018 was performed by a neurologist (H.B.) with 16 years of experience. Assessment was performed both descriptively and by means of the Natural History and Neuroprotection in Parkinson Plus Syndromes—Parkinson Plus Scale (NNIPPS‐PPS).^5^ Cognitive performance and mood status were tested with a battery of standardized neuropsychological tests and were performed by a clinical neuropsychologist (L.K.) with 16 years of experience. As impairment of multiple cognitive domains has been reported in GBM patients,^6^ the comprehensive battery comprised a wide variety of methods to assess different domains including global intellectual functioning, premorbid intellect level, language, verbal, perceptual and spatial functions, emotional and mood status, attention, and executive functions.

## Results and Discussion

The four patients evaluated were 51, 42, 32, and 31 years of age at initial diagnosis and presented with Karnofsky performance scores of 70%, 100%, 100%, and 90%, respectively. All four patients had survived until the time of this report (ie, August 2019; roughly 13–15 years), are in relatively good health, lead independent lives, and no longer receive any relevant treatment. Additional specific details regarding these patients have been published previously.^7^

As of January 2019, these patients received between 53 and 71 GBCA administrations, corresponding to 562–915 mL of a 0.5 M GBCA formulation. Increased native T_1_‐weighted SI was evident in the DN and GP of all four patients. None of the patients demonstrated any neurological or neuropsychological effects that could be attributed to GBCA administration or to Gd retention in the extrapyramidal nuclei. Two patients demonstrated no or only mild cognitive impairment, while the remaining two patients demonstrated cognitive impairment that can be attributed entirely to their age, clinical condition, and premorbid intellectual capacity. Importantly, no patient exhibited signs of rigidity, hypokinesis, or resting (or other) tremor and no patient exhibited manifestations indicative of parkinsonism or related movement disorders. Neuropsychological testing revealed no progression of tracked signs or symptoms. Although we cannot exclude selection bias in this cohort, we have no reason to hypothesize that their clinical outcome (continued survival) is related Gd retention in the brain. Our findings agree with previous observations suggesting no effect of multiple GBCA administrations on the incidence of parkinsonism^8^ and are at variance with a recent study in patients with multiple sclerosis (MS) that looked to correlate T_1_‐weighted SI increases ascribed to brain Gd retention with loss of verbal fluency.^9^ In agreement with our findings, a more recent study in patients with MS has similarly found no effect attributed to DN hyperintensity, suggestive of Gd retention, on clinical worsening as indicated by assessment of expanded disability status scale (EDSS) scores.^10^

In conclusion, multiple applications of both linear and macrocyclic GBCAs over a period of 13–15 years did not lead to clinical impairment related to Gd deposition in the DN and GP. Neurological and neuropsychological testing of our patients did not reveal any aberrant findings beyond those that could be ascribed to each patient's premorbid intellectual capacity, to the location and progression of GBM, and to the therapeutic regimens undertaken.
